# The *FGF2* gene in a myopia animal model and human subjects

**Published:** 2012-02-16

**Authors:** Jianhong An, Edward Hsi, Xiangtian Zhou, Yijin Tao, Suh-Hang Hank Juo, Chung-Ling Liang

**Affiliations:** 1 School of Optometry and Ophthalmology and Eye Hospital, Wenzhou Medical College, Wenzhou, Zhejiang, China; 2 State Key Laboratory Cultivation Base and Key Laboratory of Vision Science, Ministry of Health P.R. China and Zhejiang Provincial Key Laboratory of Ophthalmology and Optometry, Wenzhou, Zhejiang, China; 3 Department of Medical Research, Kaohsiung Medical University Hospital, Kaohsiung, Taiwan; 4 Graduate Institute of Medicine, Kaohsiung Medical University, Kaohsiung, Taiwan; 5 Department of Medical Genetics, Kaohsiung Medical University, Kaohsiung, Taiwan; 6 Bright-Eyes Clinic, Kaohsiung, Taiwan

## Abstract

**Purpose:**

Fibroblast growth factor-2 (FGF2) has been implied in the development of myopia according to previous studies investigating FGF2 in the sclera and retinal pigment epithelium. This study measured retinal *FGF2* gene expression in an animal model and also tested for the association between single nucleotide polymorphisms (SNPs) in *FGF2* and high myopia.

**Methods:**

The guinea pigs were assigned to 2 groups: form deprivation myopia (FDM) for two weeks and normal control (free of form deprivation). Biometric measurement was performed and *FGF2* expression levels were compared among the FDM eyes, the fellow eyes of the FDM group and the normal eyes in retina. We also enrolled 1,064 cases (≤-6.0 D) and 1,001 controls (≥-1.5 D) from a Chinese population residing in Taiwan. Six tagging SNPs were genotyped to test for an association between genotypes and high myopia.

**Results:**

The FDM eyes had the most prominent changes of refraction and axial length. Compared with the mRNA levels of *FGF2* in the normal eyes, the FDM eyes had the highest levels of mRNA (p=0.0004) followed by the fellow eyes (p=0.002). The FDM and normal eyes became more myopic compared with the fellow eyes, but the fellow eyes became more hyperopic (p=0.004) in the end of the experiment which may be due to its relatively short axial length when compared with normal eyes (p=0.05). The SNP genotypes were all in Hardy–Weinberg equilibrium. However, none of the SNPs were significantly associated with high myopia (all p values >0.1).

**Conclusions:**

We identified a significant change of *FGF2* expression in the FDM eyes but *FGF2* genetic variants are unlikely to influence susceptibility to myopia. There may be a systemic effect to influence gene expression and refraction on the fellow eyes, which may perturb emmetropization in the fellow eyes. Our data also suggest using normal eyes rather than the fellow eyes as the control eyes when study the form deprivation myopia.

## Introduction

Myopia is a common eye condition worldwide, and its prevalence varies widely among populations and ages [1-3]. Myopia is extremely common in Taiwan. When the definition of <-6 D is used, the prevalence of high myopia is 18% among young Taiwanese men and 24% among young Taiwanese women [3]; both of which are even higher than the 13.1% reported among young men in Singapore [2]. Furthermore, the frequency of high myopia (<-6.0 D) has increased in young Taiwanese people: 10.9% in 1983 and 21% in 2000 [4]. While studies have found several environmental risk factors, twin studies have indicated a strong genetic influence on refractive errors with estimates of heritability between 58 and 90% [5-8]. Several studies have also shown that a family history of myopia is a significant risk factor [9-13]. Recently, genetic association studies including genome-wide association studies have reported several susceptibility genes to non-syndromic myopia [14-22]. Genetic association studies are subject to the type I error, especially when the sample size is small. Therefore, replication of the genetic effects in an independent sample and the support from a functional study are important ways to reduce false positive findings.

Scleral remodeling is one of the important mechanisms for the development of myopia. In experimental myopia, eye growth is accompanied by altered proteolytic activities which could serve to remodel the structural components of the scleral extracellular matrix (ECM) [23]. Fibroblast growth factor 2 (FGF2) has been shown to be involved in the control of ECM turnover [24]. Studies have shown that exogenous delivery of FGF2 may prevent the development of myopia in chick [25]. Accordingly, FGF2 is a potential mediator of the retinoscleral signal to control scleral remodeling and ocular growth.

The first aim of the present study was to measure *FGF2* gene expression during the development of myopia in the mammals. The guinea pig model of ocular growth was used and retinal FGF2 was measured in the myopic eyes, the fellow eyes of the same animals, and the normal eyes from control animals. Given that a change of retinal *FGF2* expression was associated with myopia development in the animal study, we then tested whether genetic variants of *FGF2* were associated with high myopia in human subjects. The second aim was to test for any association between single nucleotide polymorphisms (SNPs) of *FGF2* and high myopia in a Chinese population residing in Taiwan.

## Methods

### Animal model and biometric measurement

Guinea pigs have been increasingly used as an alternative to other species in the study of myopic development [26]. All animals underwent biometric measurement before the experiment. The animal care guidelines comparable those published by the Institute for Laboratory Animal Research. The pigmented guinea pigs (three weeks old) were randomly assigned to the form deprivation myopia (FDM) group (n=14) and normal control (free of form deprivation, n=13). Animals in the FDM group wore a facemask that covered the right eye for two weeks [26]. The facemask was then removed from the animals and biometric measurement was performed in both eyes of each animal immediately. In addition, we also had the time points of biometric measurement for the normal control group matched the FDM group. Notably, we used a different inbred line of guinea pigs from what we used in the previous study [26], which led to a more efficient induction of FDM.

Biometric measures include refraction, anterior chamber depth (AC), lens thickness (LT), vitreous chamber depth (VC), and axial length (AL). The detailed measurements can be found elsewhere [26]. In brief, refraction was measured in the vertical pupil meridian by an eccentric infrared photorefractor. Since it is easy to handle guinea pigs, we could align their heads by hand until the pupil was clearly visible in the video frame. Three readings of the refractive error in the vertical meridian were recorded for each eye, and averaged data were used for further analyses. The A-scan ultrasound (AVISO Echograph class I-Type Bat; Quantel Medical, Clermont-Ferrand, France) was used to measure axial dimensions on the same day as refractions were measured. The cornea was topically anesthetized and velocities of sound were assumed as previously described [26]. Each eye was measured at least eight times, and the averages of these parameters measurements were used for analysis.

### Tissue preparation

All animal were terminated by an overdose of sodium pentobarbitone at a similar time point (between 1:00 and 3:00 PM) to minimize the effect of diurnal variation on gene expression. The eyes were enucleated and placed onto a filter paper in a Petri dish containing chilled Ringer’s solution. A circumferential incision was made along the limbus, followed by removal of the cornea, crystalline lens and vitreous body. The entire retina was separated from the choroid while the sample was soaked in iced Ringer’s solution. The retina was placed immediately into Trizol reagent (Invitrogen, Carlsbad, CA) and homogenized using the Mixer Mill MM400 (Retsch, Haan, Germany), and then moved to −80 °C before total RNA was isolated.

The animal research in this study was approved by the Animal Care and Ethics Committee at Wenzhou Medical College (Wenzhou, China). The treatment and care of animals were conducted according to the ARVO Statement for the Use of Animals in Ophthalmic and Vision Research.

### Gene expression

*FGF2* mRNA from 14 FDM eyes, 14 fellow eyes, and 26 normal eyes were measured by real-time PCR. For the normal eyes, we used the averaged expression data from both eyes of a same animal. Therefore, the sample size for normal controls was 13. Real-time PCR was run to detect the mRNA levels of *FGF2*. The PCRs were performed in an Applied Biosystems 7500 Real-Time PCR System using 2× SYBR® Green PCR Master Mix (Applied Biosystems, Foster City, CA). Total RNA was extracted from the retina with Trizol reagent (Invitrogen, Grand Island, NY) and confirmed using spectrophotometry and formaldehyde/agarose gel electrophoresis. To remove contaminating genomic DNA, 1 μg of total RNA was treated with 1 U RNase free DNase I (Promega, Madison, WI) at 37 °C for 30 min and then heated with 1 μl stop solution (Promega) at 65 °C for 10 min. Subsequently, 0.5 μg of total RNA in each sample was reversely transcribed (M-MLV reverse transcriptase; Promega) using 0.04 μg random primers (Promega) in a total volume of 20 μl according to manufacturer’s instructions. The expression level of *FGF2* mRNA was normalized to that of an internal control actin by using the equation of log_10_ (2^−ΔCt^), where ΔCt=(CT_FGF2_ – CT_actin_). The median and mean of log_10_ (2^−ΔCt^) and its standard deviation (SD) was calculated. Another internal control, 18s RNA, was used in a subset of samples to test whether different internal controls would lead to different conclusions. We used the relative expression level to indicate the fold change between different types of eyes by using the equation of 2^−ΔΔCt^. The paired *t*-test was used to compare the difference of *FGF2* expression between FDM and fellow eyes, and unpaired *t*-test was used for the data from different groups of animals.

### Genetic association study

The present study participants were enrolled from the general population with ages between 16 and 45 years. The enrollment was conducted in southern Taiwan between 2003 and 2009. All the participants were of Chinese descents. All the cases had myopia in both eyes and had a spherical refraction ≤-6.0 D in at least one eye. A subject with a spherical refraction ≥-1.5 D in the more myopic eye was defined as a control. We used negative cylindrical powers in all subjects. In addition, none of the controls had received any previous refractive surgery. The refractive error was measured without cycloplegia for subjects with ages ≥18 years and with cycloplegia with ages <18 years. The refractive error was measured using autorefractometers (Topcon KR-8100 or RM-8800; Topcon, Tokyo, Japan) for all eyes. A written informed consent was given by each subject or custodian (if the age of the participant was less than 18 years old). The study was approved by the Institutional Review Board at the Kaohsiung Medical University Hospital, Taiwan. The research followed the tenets of the Declaration of Helsinki.

### SNP selection and genotyping

We first selected the tagging single nucleotide polymorphisms (tSNPs) at the *FGF2* gene from the release 3.0 Phase II data of the HapMap Project using the Tagger Pairwise method [27]. tSNPs were chosen according to the following criteria: r^2^≥0.8 and the minor allele frequency (MAF) ≥10% in the Han Chinese population. A total of six tSNPs met the selection criteria, which were rs308442 (intron 1), rs17473132 (intron 1), rs308379 (intron 1), rs308381 (intron 1), rs1048201 (3′UTR), and rs3804158 (3′UTR). Genotyping was performed by using TaqMan technology. Briefly, PCR primers (Table 1) and TaqMan minor groove binder (MGB) probes were designed and reactions were performed in 96-well microplates with ABI 9700 thermal cyclers (Applied Biosystems, Foster City, CA). The condition to run real-time PCR was as follows: 50 °C for 2 min; 95 °C for 10 min; 95 °C for 15 s; 60 °C for 1 min; the last 2 steps repeated for 45 cycles. Fluorescence was measured with the ABI 7900 Real Time PCR System (Applied Biosystems) and analyzed with its System SDS software version 1.2.3.

**Table 1:** The kit assay ID for each SNP.

**SNP ID**	**Assay ID**	**Forward primer name**	**Reverse primer name**
rs3804158	C__27486143_10	C__27486143_10_F	C__27486143_10_R
rs1048201	C___8837778_10	C___8837778_10_F	C___8837778_10_R
rs308442	C____802937_10	C____802937_10_F	C____802937_10_R
rs308379	C____802931_10	C____802931_10_F	C____802931_10_R
rs308381	C____802929_20	C____802929_20_F	C____802929_20_R
rs17473132	C__34186735_10	C__34186735_10_F	C__34186735_10_R

### Statistical analysis for genetic polymorphism studies

The allele frequency was obtained by direct gene counting. Hardy–Weinberg equilibrium (HWE) was tested in controls [28] by using the χ^2^ test for each SNP. According to the myopic status and three genotypes of each SNP, the χ^2^ test for a 2×3 contingency table or Fisher exact test was performed. The genotype specific odds ratio (OR) was first checked to test for the allele dominance. If one allele is dominant over the other allele, the two genotypes containing the dominant allele would be combined to increase the statistical power. When there is no evidence of dominance, we prefer to not collapse heterozygotes with minor homozygotes unless the number of minor homozygotes is too small.

## Results

### Confirmation of phenotypic changes induced by form deprivation

The refractions of the guinea pig eyes in the two groups indicated hyperopia before the experiment (3 weeks of age). No significant differences of refraction, or AL, AC, LT, or VC (by the paired *t*-test) between the two eyes of a same animal (Table 2 and Table 3) at the beginning of the experiments. The FDM eyes became more myopic by 4.32 D and had an increase of axial length by 0.37 mm after form deprivation for two weeks (Table 2). The refraction data showed the normal eyes also had a myopic shift in two weeks, which may reflect physiologic emmetropization. Unexpectedly, the fellow eyes became more hyperopic when compared with the refraction shift in the normal control eyes. The average increase of AL was 0.21 mm in the fellow eyes and 0.25 mm in the normal eyes (Table 2). The largest increase of eye component in the FDM eyes was in VC (Table 3). On the contrary, the average VC was not increased in the fellow eyes of the FD group in the end of the experiments, while the normal eyes had increased VC (Table 3). Other parameters such as AC and LT had no significant differences between the two eyes of a same animal after form deprivation for two weeks.

**Table 2:** Refraction and axial length (AL) data measured in the beginning and end of the experiments in the three types of eyes.

** **	** **	**Refraction (D)**				**AL (mm)**			
** **	** **	**CD group (n=14)**		**Normal (n=13)**		**FD group (n=14)**		**Normal (n=13)**	
**Week**	**Type of eye**	**FDM**	**Fellow**	**L**	**R**	**FDM**	**Fellow**	**L**	**R**
0	Mean±SD	6.14± 1.15	6.44±1.02	6.51±0.98	6.34±1.05	7.81±0.11	7.81±0.11	7.87±0.11	7.87±0.11
	p	0.57		0.06		0.95		0.89	
2	Mean±SD	1.82±2.33	7.29±0.77	6.03±0.90	6.08±0.92	8.18±0.17	8.02±0.11	8.11±0.13	8.12±0.14
	p	<0.001		0.77		<0.001		0.52	

FD: form deprivation; FDM: form deprivation myopia. P values are the comparison between the two eyes of a same animal using the paired *t*-test.

**Table 3:** The length of each eye component. The data are presented as mean±SD.

	**AC (mm)**				**LT (mm)**				**VC (mm)**			
	**FD group (n=14)**		**Normal (n=13)**		**FD group (n=14)**		**Normal (n=13)**		**FD group (n=14)**		**Normal (n=13)**	
**Type of eye **	**FDM**	**Fellow**	**L**	**R**	**FDM**	**Fellow**	**L**	**R**	**FDM**	**Fellow**	**L**	**R**
Wk 0	1.01±0.04	1.02±0.04	1.04±0.04	1.03±0.04	3.71±0.08	3.69±0.07	3.72±0.07	3.72±0.08	3.10±0.07	3.10±0.06	3.12±0.08	3.12±0.08
p value	0.10		0.15		0.07		0.46		0.84		0.13	
Wk 2	1.05±0.03	1.04±0.04	1.05±0.04	1.05±0.04	3.88±0.07	3.89±0.07	3.90±0.10	3.89±0.10	3.23±0.13	3.09±0.06	3.16±0.11	3.16±0.09
p value	0.22		0.37		0.38		0.30		<0.001		0.80	

FD: form deprivation; FDM: form deprivation myopia. P values are the comparison between the two eyes of a same animal using the paired *t*-test.

### Gene expression

**Figure 1:**
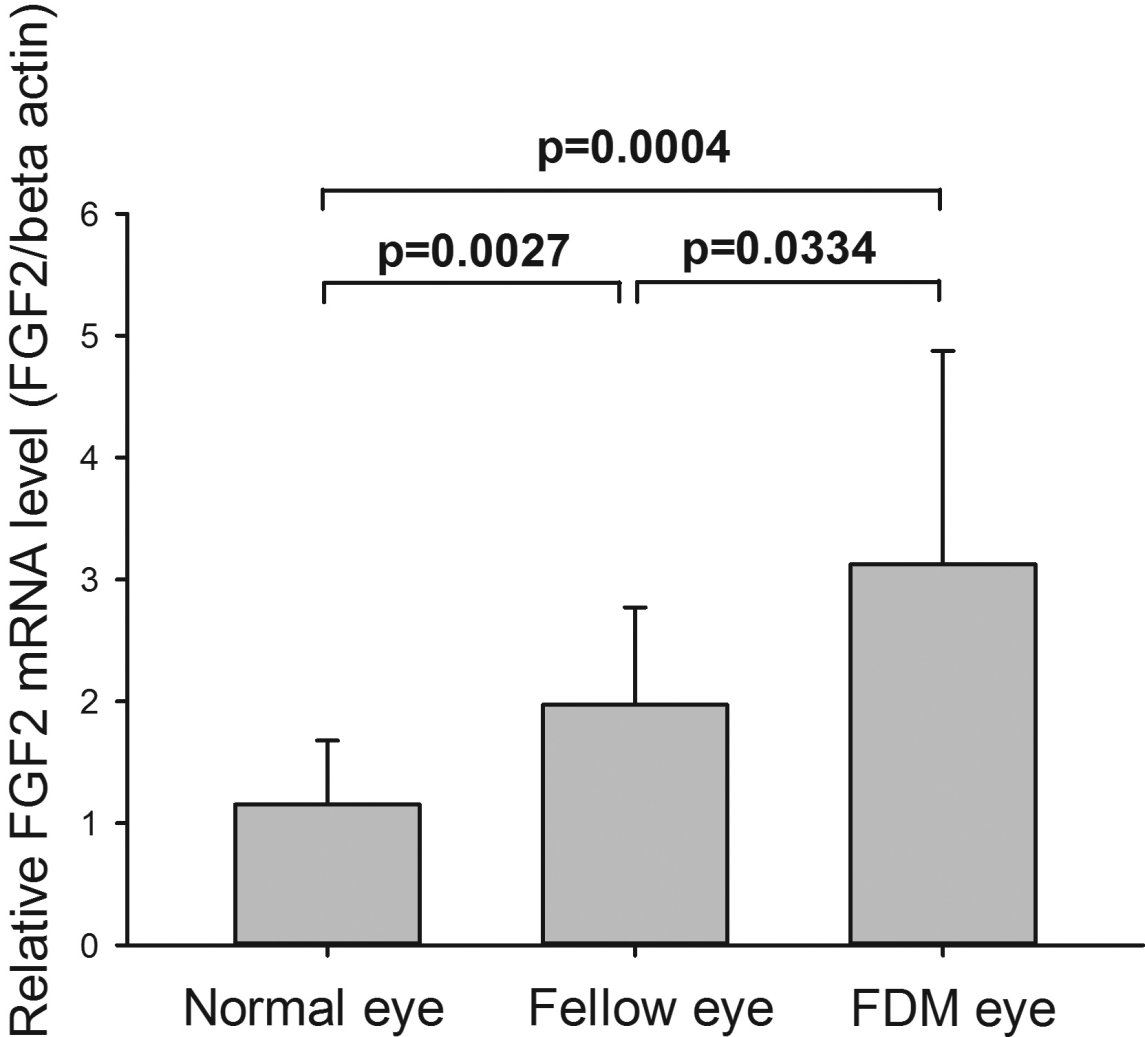
Relative *FGF2* mRNA level in FDM, fellow and normal eyes. The expression levels of FDM eye (n=14) and fellow eye (n=14) were significantly higher than normal eye (n=13, the averaged data from both eyes was used for each animal; p=0.00004 and 0.0027, respectively). The expression level of FDM eye was also higher than the fellow eye (p=0.0334). The expression level of normal eye was the expression level of right (n=13) eyes of normal control guinea pigs. The test of significance in comparing FDM and fellow eyes was using paired-t statistics, and unpaired *t*-test for the comparison between eyes of normal controls and eyes of form deprivation group.

The mRNA levels of *FGF2* were highest in the FDM eyes, followed by the fellow eyes of the FDM group and lowest in the eyes of the normal controls that were free of form deprivation. This pattern was present no matter actin or 18s RNA was used as the internal control. The differences of the mRNA levels between any two groups are shown in Figure 1. Using the data in the normal eyes as the reference, mRNA levels in the fellow eyes of the FDM group was increased by 1.7 fold (p=0.0027), and by 2.7 fold (p=0.0004) in the FDM eyes. The difference between FDM and fellow eyes of a same animal was also significant (by 1.6 fold, p=0.033).

### Genetic association study

A total of 1,064 cases and 1,001 controls were included in the present study. The mean age was 21.8 years for cases and 21.6 years for controls. The spherical refractions ranged from −6.0 D to −23.5 D with a mean of −8.0 D and SD of 1.8 D for cases. For the controls, the spherical refractions ranged from 0.75 D to −1.5 D, and the mean±SD was −0.4±0.6 D. The call rate for the 6 SNPs ranged from 93% to 97%.

The frequencies of the genotypes and the associations between high myopia (≤-6.0 D) and the six tSNPs are shown in Table 4. All the SNPs were in HWE in the controls. There was no significant difference of either allele frequency (data not shown) or genotype frequency (Table 4) between cases and controls. We further performed exploratory analysis to compare extreme myopia (≤-10 D) versus more stringent controls (≥-0.5). Only SNP, rs308379, had a nominal p value=0.027 for the re-defined cases and controls **(**Table 5**)**. However, this significant p value was mainly driven by the over-represented heterozygotes in the cases (the genotype distributions of AA, AT, and TT were 26.7%, 58.8%, and 14.5% in the cases; 35.4%, 45.9%, and 18.8% in the controls). Notably, the T allele exerted as a risk allele in the AT genotype but such a detrimental effect disappeared in the TT genotype. Furthermore, the p value was not significant any more after the multiple testing correction by either the Bonferroni method (p=0.32 after the Bonferroni correction) or permutation test (p=0.23). Accordingly, we failed to show any association between *FGF2* SNPs and high myopia or extreme myopia.

**Table 4:** The association between tSNPs and their relationships to high myopia (refraction in case ≤-6 D; in control ≥-1.5D).

**SNP (major/minor)**	**Status**	**Major homozygote**	**Heterozygote**	**Minor homozygote**	**MAF**	**p value**
rs308442	Case	642 (64.8%)	317 (32.0%)	31 (3.1%)	19.1%	0.2293
(T/A)	Control	620 (63.7%)	308 (31.7%)	45 (4.6%)	20.5%	
rs17473132	Case	930 (88.6%)	118 (11.2%)	2 (0.2%)	5.8%	0.9119*
(G/A)	Control	877 (88.5%)	111 (11.2%)	3 (0.3%)	5.9%	
rs308379	Case	348 (33.2%)	516 (49.1%)	186 (17.7%)	42.3%	0.6037
(A/T)	Control	335 (33.8%)	467 (47.1%)	189 (19.1%)	42.6%	
rs308381	Case	991 (92.3%)	81 (7.6%)	1 (0.1%)	3.9%	0.4931*
(T/C)	Control	940 (93.3%)	65 (6.5%)	2 (0.2%)	3.4%	
rs1048201	Case	260 (24.4%)	532 (50.0%)	272 (25.6%)	50.6%	0.2736
(C/T)	Control	245 (24.5%)	529 (52.8%)	227 (22.7%)	49.1%	
rs3804158	Case	366 (34.8%)	497 (47.3%)	188 (17.9%)	41.5%	0.5546
(G/A)	Control	360 (36.1%)	477 (47.8%)	161 (16.1%)	40.0%	

P values were from 2×3 Tables (3 genotypes versus 2 phenotypes). *p-value is adjusted by fisher exact test; MAF: minor allele frequency.

**Table 5:** Six tSNPs and their relationships to extreme myopia (refraction in case ≤-10 D; in control ≥-0.5D).

**SNP (major/minor)**	**status**	**Major homozygote**	**Heterozygote**	**Minor homozygote**	**MAF**	**p value**
rs308442	Case	76 (66.1%)	35 (30.4%)	4 (3.5%)	18.7%	0.7511*
(T/A)	Control	363 (62.7%)	187 (32.3%)	29 (5.0%)	21.2%	
rs17473132	Case	118 (92.2%)	10 (7.8%)	0 (0.0%)	3.9%	0.4028*
(G/A)	Control	520 (88.3%)	68 (11.5%)	1 (0.2%)	5.9%	
rs308379	Case	35 (26.7%)	77 (58.8%)	19 (14.5%)	43.9%	0.0276
(A/T)	Control	209 (35.4%)	271 (45.9%)	111 (18.8%)	41.7%	
rs308381	Case	123 (93.9%)	8 (6.1%)	0 (0.0%)	3.1%	0.8645
(T/C)	Control	560 (93.5%)	39 (6.5%)	0 (0.0%)	3.3%	
rs1048201	Case	28 (21.4%)	72 (55.0%)	31 (23.7%)	51.1%	0.784
(C/T)	Control	144 (24.2%)	314 (52.8%)	137 (23.0%)	49.4%	
rs3804158	Case	47 (36.2%)	64 (49.2%)	19 (14.6%)	39.2%	0.9697
(G/A)	Control	216 (36.4%)	286 (48.2%)	91 (15.4%)	39.5%	

*p-value is adjusted by fisher exact test.

## Discussion

We used the form deprivation to induce myopia in the guinea pigs and compared retinal FGF2 expression levels among the FDM eyes, fellow eyes of the experimental animals and normal eyes of the control animals. The covered eyes had the highest expression level, followed by the uncovered fellow eyes of the same animals, and eyes of control animals had the lowest expression levels. This result indicates that FGF2 overexpression in the retina may beassociated with the development of form deprivation myopia. We noticed that the fellow eyes had a tendency of hyperopia while the normal eyes in the control group had a myopic shift. From both the FGF2 expression data and the change of refraction, we speculated that form deprivation may interrupt emmetropization in both eyes of animals and also the physiologic control of gene expression. We also tested for the association between six tSNPs at the FGF2 gene and high myopia in a Chinese population residing in Taiwan. However, the genetic association study failed to show any significant results. To ensure we would not miss the genetic effect that only exists for extremely high myopia as we saw before [20], we also performed analysis for extreme myopia in comparison with stringently defined controls. This approach also did not reveal any significant associations when a correction of multiple testing was taken into account. Therefore, even though FGF2 may play a role in the myopia development, its genetic polymorphisms are unlikely to influence inter-individual susceptibility to high myopia.

It is generally accepted that the development of myopia is based on the local control as hemi-retinal occlusion leads to only hemiocular elongation [29]. Therefore, the contralateral fellow eye of an animal that receives experimentally induced myopia has been widely used as a control for myopia studies. In the present study, we showed that *FGF2* expression was also influenced in the fellow eyes as well as biometric data (especially VC). Since all fellow eyes in the 14 guinea pigs had increased refractive powers (p=0.004) rather than emmetropic shift, we hypothesized that the physiologic emmetropization in the fellow eyes was disturbed. This hyperopic change is probably due to failure of an increase of VC. An increase of *FGF2* expression along with an increase of refractive power in the fellow eyes further indicates the possibility of perturbed eye development when their contra-lateral eyes are under form deprivation. Previous studies using either three shrews [30] or guinea pigs [31] also demonstrated a relatively hyperopic shift in the fellow eyes of the induced-myopia animals. The above data suggest that independent control animals without any vision manipulations are necessary for a better experimental animal study.

The relationship between the *FGF2* expression pattern and myopia is inconsistent among previous studies. The scleral and retinal levels of FGF2 have been reported as no difference between the myopic and fellow eyes in the tree shrew receiving induced myopia [32]. However, their data showed a decrease of mean *FGF2* expression in the anterior sclera of myopic eyes than in the normal control eyes although the difference of FGF2 levels did not reach a significant level. Furthermore, the same study [32] demonstrated an upregulation of the FGF2 receptor in the myopic eyes. A recent study using the human scleral tissue showed that atropine increased *FGF2* activation in a dose-dependent manner [33], while the authors also reported atropine reduced cell proliferation of scleral fibroblasts. Since atropine has been demonstrated to retard myopia progression in humans [34,35], their *FGF2* expression pattern is unexpected and hard to explain (personal communication with the correspondent author of the study [33]). Similar to our finding, *FGF2* was significantly upregulated in the choroid/RPE of minus lens-treated eyes (i.e., eyes of induced myopia) of primate marmoset monkeys as compared with plus lens-treated fellow eyes (i.e., eyes of induced hyperopia) [36].

Previous studies also reported that the refraction and gene expression of fellow eyes can be influenced by the treated eyes [37]. Consistent with the previous study [37], our data showed that the major difference of eye component between fellow eyes and FDM eyes was VC. The hyperopic shift in our data may be due to the lack of growth of VC in the fellow eyes (Table 3). The amount of increased AL was largest in the FDM eyes, followed by normal eyes and then the fellow eyes. However, Frost et al. [37] reported the order of increased AL as treated eyes, fellow eyes and normal eyes in their lens-induced myopia in tree shrews. Accordingly, the “cross-over” effect on the fellow eyes may be species specific or model specific.

Although animal studies have revealed *FGF2* as a candidate gene, the relationship between genetic polymorphisms of *FGF2* and myopia has not been extensively investigated. Tsai et al. [38] examined two SNPs (one is in the promoter and the other in the 3′ UTR) at the *FGF2* gene and reported no association with high myopia in a Chinese population. Mutti et al. [39] also reported no linkage disequilibrium between *FGF2* and myopia using the TDT statistical program in a family data. It needs to be noticed that the failure to identify significant SNPs at the *FGF2* gene does not exclude the importance of this gene in the pathogenesis of myopia. Instead, the results of genetic association study should be interpreted as the genetic variants at the *FGF2* gene may not influence individual susceptibility to high myopia. Namely, the genetic variants of this gene can not serve as a biomarker to predict a risk for high myopia. Our sample size provided a power of 83% to detect a common polymorphism with an OR of 1.25. Therefore, our conclusion is unlikely to be biased by the sample size or genetic effect issues.

In conclusion, we identified a significant change of *FGF2* expression levels in the FDM eyes of the guinea pigs. The refractive data indicated that a disturbance of emmetropization of the fellow eyes resulted in their hyperopic shift. Therefore, we suggest using normal eyes rather than the fellow eyes of same animals as the control eyes because form deprivation may have a systemic effect to cause alteration in the gene expression and refraction in the fellow eyes. Our findings may have an important clinical implication in that clinicians need to watch out the change of the fellow eyes while treating children of vision impairment in one eye. None of the tested SNPs are significantly associated with high myopia, which suggests that *FGF2* genetic variants are unlikely to influence individual difference of myopia susceptibility.

## References

[1] Katz J, Tielsch JM, Sommer A (1997). Prevalence and risk factors for refractive errors in an adult inner city population.. Invest Ophthalmol Vis Sci.

[2] Wu HM, Seet B, Yap EP, Saw SM, Lim TH, Chia KS (2001). Does education explain ethnic differences in myopia prevalence? A population-based study of young adult males in Singapore.. Optom Vis Sci.

[3] Lin LL, Shih YF, Hsiao CK, Chen CJ, Lee LA, Hung PT (2001). Epidemiologic study of the prevalence and severity of myopia among schoolchildren in Taiwan in 2000.. J Formos Med Assoc.

[4] Lin LL, Shih YF, Hsiao CK, Chen CJ (2004). Prevalence of myopia in Taiwanese schoolchildren: 1983 to 2000.. Ann Acad Med Singapore.

[5] Hammond CJ, Snieder H, Gilbert CE, Spector TD (2001). Genes and environment in refractive error: the twin eye study.. Invest Ophthalmol Vis Sci.

[6] Teikari JM, Kaprio J, Koskenvuo MK, Vannas A (1988). Heritability estimate for refractive errors–a population-based sample of adult twins.. Genet Epidemiol.

[7] Teikari JM, O'Donnell J, Kaprio J, Koskenvuo M (1991). Impact of heredity in myopia.. Hum Hered.

[8] Lyhne N, Sjolie AK, Kyvik KO, Green A (2001). The importance of genes and environment for ocular refraction and its determiners: a population based study among 20–45 year old twins.. Br J Ophthalmol.

[9] Liang CL, Yen E, Su JY, Liu C, Chang TY, Park N, Wu MJ, Lee S, Flynn JT, Juo SH (2004). The impact of the family history of high myopia on level and onset of myopia.. Invest Ophthalmol Vis Sci.

[10] Goss DA, Jackson TW (1996). Clinical findings before the onset of myopia in youth: 4. Parental history of myopia.. Optom Vis Sci.

[11] Zadnik K, Satariano WA, Mutti DO, Sholtz RI, Adams AJ (1994). The effect of parental history of myopia on children's eye size.. JAMA.

[12] Mutti DO, Mitchell GL, Moeschberger ML, Jones LA, Zadnik K (2002). Parental myopia, near work, school achievement, and children's refractive error.. Invest Ophthalmol Vis Sci.

[13] Wu MM, Edwards MH (1999). The effect of having myopic parents: an analysis of myopia in three generations.. Optom Vis Sci.

[14] Hammond CJ, Andrew T, Mak YT, Spector TD (2004). A susceptibility locus for myopia in the normal population is linked to the PAX6 gene region on chromosome 11: a genomewide scan of dizygotic twins.. Am J Hum Genet.

[15] Han W, Leung KH, Fung WY, Mak JY, Li YM, Yap MK, Yip SP (2009). Association of PAX6 polymorphisms with high myopia in Han Chinese nuclear families.. Invest Ophthalmol Vis Sci.

[16] Inamori Y, Ota M, Inoko H, Okada E, Nishizaki R, Shiota T, Mok J, Oka A, Ohno S, Mizuki N (2007). The COL1A1 gene and high myopia susceptibility in Japanese.. Hum Genet.

[17] Liang CL, Hung KS, Tsai YY, Chang W, Wang HS, Juo SH (2007). Systematic assessment of the tagging polymorphisms of the COL1A1 gene for high myopia.. J Hum Genet.

[18] Hall NF, Gale CR, Ye S, Martyn CN (2009). Myopia and polymorphisms in genes for matrix metalloproteinases.. Invest Ophthalmol Vis Sci.

[19] Liang CL, Wang HS, Hung KS, Hsi E, Sun A, Kuo YH, Juo SH (2006). Evaluation of MMP3 and TIMP1 as candidate genes for high myopia in young Taiwanese men.. Am J Ophthalmol.

[20] Liang CL Hsi E Chen KC Pan YR Wang YS Juo SH A functional polymorphism at 3′ UTR of the PAX6 gene may confer risk for extreme myopia in Chinese. Invest Ophthalmol Vis Sci 2011 52 3500 5 21421876 10.1167/iovs.10-5859

[21] Solouki AM, Verhoeven VJ, van Duijn CM, Verkerk AJ, Ikram MK, Hysi PG, Despriet DD, van Koolwijk LM, Ho L, Ramdas WD, Czudowska M, Kuijpers RW, Amin N, Struchalin M, Aulchenko YS, van Rij G, Riemslag FC, Young TL, Mackey DA, Spector TD, Gorgels TG, Willemse-Assink JJ, Isaacs A, Kramer R, Swagemakers SM, Bergen AA, van Oosterhout AA, Oostra BA, Rivadeneira F, Uitterlinden AG, Hofman A, de Jong PT, Hammond CJ, Vingerling JR, Klaver CC (2010). A genome-wide association study identifies a susceptibility locus for refractive errors and myopia at 15q14.. Nat Genet.

[22] Hysi PG, Young TL, Mackey DA, Andrew T, Fernandez-Medarde A, Solouki AM, Hewitt AW, Macgregor S, Vingerling JR, Li YJ, Ikram MK, Fai LY, Sham PC, Manyes L, Porteros A, Lopes MC, Carbonaro F, Fahy SJ, Martin NG, van Duijn CM, Spector TD, Rahi JS, Santos E, Klaver CC, Hammond CJ (2010). A genome-wide association study for myopia and refractive error identifies a susceptibility locus at 15q25.. Nat Genet.

[23] Jones BE, Thompson EW, Hodos W, Waldbillig RJ, Chader GJ (1996). Scleral matrix metalloproteinases, serine proteinase activity and hydrational capacity are increased in myopia induced by retinal image degradation.. Exp Eye Res.

[24] Pickering JG, Ford CM, Tang B, Chow LH (1997). Coordinated effects of fibroblast growth factor-2 on expression of fibrillar collagens, matrix metalloproteinases, and tissue inhibitors of matrix metalloproteinases by human vascular smooth muscle cells. Evidence for repressed collagen production and activated degradative capacity.. Arterioscler Thromb Vasc Biol.

[25] Rohrer B, Stell WK (1994). Basic fibroblast growth factor (bFGF) and transforming growth factor beta (TGF-beta) act as stop and go signals to modulate postnatal ocular growth in the chick.. Exp Eye Res.

[26] Zhou X, Ye J, Willcox MD, Xie R, Jiang L, Lu R, Shi J, Bai Y, Qu J (2010). Changes in protein profiles of guinea pig sclera during development of form deprivation myopia and recovery.. Mol Vis.

[27] Barrett JC, Fry B, Maller J, Daly MJ (2005). Haploview: analysis and visualization of LD and haplotype maps.. Bioinformatics.

[28] Xu J, Turner A, Little J, Bleecker ER, Meyers DA (2002). Positive results in association studies are associated with departure from Hardy-Weinberg equilibrium: hint for genotyping error?. Hum Genet.

[29] Fredrick DR (2002). Myopia.. BMJ.

[30] McBrien NA, Norton TT (1992). The development of experimental myopia and ocular component dimensions in monocularly lid-sutured tree shrews (Tupaia belangeri).. Vision Res.

[31] Ren Y, Xie R, Zhou X, Pan M, Lu F (2011). Spontaneous high myopia in one eye will affect the development of form deprivation myopia in the fellow eye.. Curr Eye Res.

[32] Gentle A, McBrien NA (2002). Retinoscleral control of scleral remodelling in refractive development: a role for endogenous FGF-2?. Cytokine.

[33] Barathi VA, Weon SR, Beuerman RW (2009). Expression of muscarinic receptors in human and mouse sclera and their role in the regulation of scleral fibroblasts proliferation.. Mol Vis.

[34] Saw SM, Gazzard G, Au Eong KG, Tan DT (2002). Myopia: attempts to arrest progression.. Br J Ophthalmol.

[35] Tong L, Huang XL, Koh AL, Zhang X, Tan DT, Chua WH (2009). Atropine for the treatment of childhood myopia: effect on myopia progression after cessation of atropine.. Ophthalmology.

[36] Shelton L, Troilo D, Lerner MR, Gusev Y, Brackett DJ, Rada JS (2008). Microarray analysis of choroid/RPE gene expression in marmoset eyes undergoing changes in ocular growth and refraction.. Mol Vis.

[37] Frost MR, Norton TT (2012). Alterations in Protein Expression in Tree Shrew Sclera during Development of Lens-induced Myopia and Recovery.. Invest Ophthalmol Vis Sci.

[38] Lin HJ, Wan L, Tsai Y, Liu SC, Chen WC, Tsai SW, Tsai FJ (2009). Sclera-related gene polymorphisms in high myopia.. Mol Vis.

[39] Mutti DO, Cooper ME, O'Brien S, Jones LA, Marazita ML, Murray JC, Zadnik K (2007). Candidate gene and locus analysis of myopia.. Mol Vis.

